# Combining Immersive Simulation with a Collaborative Procedural Training on Local Anesthetic Systemic Toxicity and Fascia Iliaca Compartment Block: A Pilot Study

**DOI:** 10.5811/westjem.25020

**Published:** 2025-01-15

**Authors:** Katherine B. Griesmer, Maxwell Thompson, Briana Miller, Guihua Zhai, Jaron Raper, Andrew Bloom

**Affiliations:** * University of Alabama at Birmingham Heersink School of Medicine, Department of Emergency Medicine, Birmingham, Alabama; † University of Alabama at Birmingham, CCTS Biostatistics, Epidemiology & Research Design (BERD), Birmingham, Alabama

## Abstract

**Introduction:**

Readiness to perform a wide variety of procedures or manage nearly any patient presentation remains an essential aspect of emergency medicine training and practice. Often, simulation is needed to supplement real-life exposure to provide comfort and knowledge, particularly with rarer pathology and procedures. As the scope of practice continues to grow, newer procedures, such as ultrasound (US)-guided nerve blocks (UGNB), are becoming integrated into resident training, building on previously established skills. The fascia iliaca compartment block (FICB) is performed on patients with specific femoral fractures and is a now a component of standard multimodal pain regimens, with US-guidance limiting adverse events. Given the need for high volumes of local anesthetic to perform the block it is imperative for clinicians to understand dosing as well as recognize and treat local anesthetic systemic toxicity (LAST). With sparse literature on sequential immersive and procedural simulation involving intertwined topics, this presents a unique opportunity for learners.

**Methods:**

To study the perceived knowledge and comfort with FICB and LAST, a pilot study was developed with two separate but concurrent one-hour simulations completed encompassing one of each topic over one day. We surveyed 19 learners, consisting of residents ranging from postgraduate years 1–3, prior to and immediately following completion, regarding their perceptions. We used the Stuart-Maxwell test to compare survey data.

**Results:**

More than half of participants (56%) had not received prior formal training on FICB. There was a positive trend in perceived confidence and knowledge with visualizing relevant anatomy (4.0 [2.0–6.0] vs 9.0 [7.5–10.0], *P* = 0.10), performing FICB (4.0 [1.0–5.0] vs 9.0 [7.0–10.0, *P* = 0.08]), and perceived ability to teach their peers (3.0 [1.0–5.0] vs 8.5 [7.0–10.0], *P* = 0.20). Perceived ability in diagnosing and managing LAST also increased following the simulation (5.0 [3.0–6.0] vs 6.0 [6.0–7.0], *P* = 0.12 and 3.0 [2.0–6.0] vs 6.0 [6.0–7.0], *P* = 0.08, respectively).

**Conclusion:**

Learners’ perceptions of this simulation experience echo the findings of previous studies in which simulation can be used to teach procedures and pathology; of note, however, we presented a novel experience with a combination of immersive and procedural simulation.

## INTRODUCTION

To enhance preparedness to care for uncommon patient presentations and procedures, simulation has often been used in graduate medical education (GME) to lay the foundation as well as to fine tune skills, including resuscitations.^1^ Simulation has also been at the forefront of procedural skill acquisition and increasing learner’s confidence at both the GME and undergraduate medical education levels, with a focus on both high-acuity and low-occurrence procedures.^2^ ^–^ ^4^ Both in simulation and clinical practice, more procedures are being performed under ultrasound (US) guidance with trends toward increased safety and efficacy, most notably including placement of central venous lines.^5^ ^,^ ^6^ With this increasing incorporation of US training in emergency medicine (EM) residency programs, the variety of procedures performed in an emergency department (ED) setting have also expanded.

Regional nerve blocks were previously completed via landmark only; however, there has been progression toward US-guidance due to reduced adverse events and greater first-attempt success rates.^7^ ^,^ ^8^ Regional nerve blocks have increasingly fallen under the scope of EM practice, particularly with regard to US-guided nerve blocks (UGNB). Many patients are poor candidates for frequent or high doses of opioids as a primary pain management strategy in the ED.^9^ One study found almost 25% of elderly patients suffered from delirium while hospitalized, with the majority of those receiving polypharmacy.^10^ However, UGNB is a valuable tool for managing pain in hip fractures, regardless of patient ability to tolerate opioid and non-opioid analgesics. Nerve blocks are now recommended by the American College of Surgeons and the American Academy of Orthopaedic Surgeons as a standard component of a multimodal approach to pain management.^11^ ^,^ ^12^ The American College of Emergency Physicians (ACEP) recently stated that UGNBs make up a core skill for emergency physicians, voicing broad support for its use and citing its versatility for a variety of procedures, from complex laceration repairs to orthopedic reductions/splinting.^7^

The fascia iliaca compartment block (FICB) can provide significant analgesia, particularly in populations that may have contraindications or comorbidities that preclude standard systemic intravenous and/or oral pain regimens including opioids, ketorolac, and other analgesic agents. The block, with its discovery in 1989 and eventual introduction in the EM literature in 2007, has been slow to be adopted despite its safety profile and efficacy.^13^ ^,^ ^14^ The FICB can be used for femoral neck fractures in the pre-, peri-, and postoperative stages given the blockade of femoral nerve, local femoral cutaneous nerve, and variable coverage of the obturator nerve.^8^ ^,^ ^15^ By incorporating US guidance, the compartment block is done lateral to the femoral triangle (femoral nerve, artery, and vein), thus minimizing the chance of intravascular injection.^13^ A meta-analysis of FICB has also been shown to reduce morphine dosing requirement and may even negate the need for additional medications beyond the block.^16^ In another study, 90% of patients had blockade with a significant reduction in the visual analogue scale from 7.5 to 2.94 at the 20-minute mark.^17^ With such compelling data regarding its efficacy and the widespread availability of US in EDs, the FICB represents a powerful tool in pain control in the ED setting that is well within the scope of the emergency physician. Within our current practice, this could result in increased patient satisfaction and possibly free up more resources, including nursing, particularly if not requiring consistent treatment for breakthrough pain.

Local anesthetics are commonplace within the medical field and especially within the ED where they are a routine component of any clinician’s medical practice.^18^ However, complications exist, particularly when large quantities of anesthetic are used, or inadvertent intravascular injection occur, which may cause local anesthetic systemic toxicity (LAST). Elderly patients or those with organ dysfunction are at a particularly high risk.^19^ ^,^ ^20^ Further complicating the syndrome, LAST has considerable variability in onset and symptomatology.^19^ It can be detrimental through its effects on both the central nervous system (CNS) and cardiovascular systems, resulting in arrhythmias, seizures, cardiovascular collapse, and risk of cardiac arrest. Each anesthetic agent has its own maximum, weight-based dosing that may be augmented if formulated in combination with epinephrine.^19^

Previously, it was believed the agents would behave in a predictable, stepwise manner with precedent CNS symptoms appearing prior to cardiac dysrhythmias; however, the more potent agents have been found to have preceding and possibly concomitant cardiac and CNS toxicity.^20^ The incidence of LAST is variable with one study reporting occurrence in up to 25 per 10,000 blockades and another specifying occurrence in 79 of 10,000 brachial plexus blockades.^20^ Regardless of the true incidence, LAST occurrence has been shown to be reduced with US-guided regional anesthesia by up to 65%, although risk still exists.^21^ Thus, training on recognition of the signs and symptoms, as well as treatment, is imperative for emergency physicians. Simulation of this procedure and its most dangerous complication allows learners an opportunity to gain experience with the condition without effects on patient outcomes. Following recognition of LAST, injection must be first discontinued and in severe cases may require administration of intralipid emulsion therapy (ILE). The American Heart Association also includes ILE in its guidelines for cardiac arrest secondary to LAST.^22^

Following ACEP guidelines and expanding on basics of well-known procedures, FICB may be a beneficial procedure for emergency physicians to master along with the consideration of the risks of LAST and its management. Simulation has been documented as being an effective teaching modality, offering a safe environment for learners.^23^ ^,^ ^24^ Ultrasound-based training has been previously shown to be beneficial with an improvement in confidence and procedural skills.^23^ The literature is sparse on a combination of sequential immersive and procedural simulation techniques in medical education. While taking into consideration the variable presentations and severity of complications related to LAST, as well as the rising importance of regional nerve blocks in EM, a paired simulation experience can improve identification and treatment of the syndrome, as well as allow learners to enhance their skillset. Here we present a pilot study on EM residents’ perceptions and confidence with diagnosing and managing LAST as well as procedural skills with FICB.

## OBJECTIVES

Our objective in this study was to create an immersive simulation that teaches EM residents to recognize clinical signs and symptoms of LAST, develop an appropriate treatment algorithm, and manage potential outcomes including cardiac arrest. Secondary objectives included successful performance of US-guided FICB, troubleshooting complications, and determining proper local anesthetic dosing to prevent LAST. Ultimately, the goal was to develop a simulation-based curriculum to increase resident comfort and knowledge with the FICB while recognizing and managing its rare and more dangerous complications.

## METHODS

We conducted a prospective pilot study, deemed exempt by the institutional review board, for both an immersive case and procedural simulation in the fall of 2023. A pre-simulation questionnaire was administered a month prior with a focus on residents’ perceived comfort levels with various US-guided procedures, along with uncommon causes of cardiac arrest and their management. Another pre-questionnaire was administered just prior to the procedural FICB simulation regarding comfort and knowledge with the specific procedure.

Participants were EM residents ranging from postgraduate years (PGY) 1–3 at a Level I trauma center university hospital system. A convenience sample of 19 residents who were present for the conference day participated. All participants voluntarily agreed to participate in the activities with informed consent provided. As part of the residency curriculum, residents must complete a four-week rotation focusing on US skills and interpretation as PGY-1s as well as fulfill the Accreditation Council for Graduate Medical Education-required number of resuscitations, ultrasounds, and procedures. An additional four weeks of the PGY-1 year is devoted to toxicology with focus on awareness and management of toxicologic emergencies. However, as the session was completed in the first half of the year, not all PGY-1s had completed a toxicology and/or US rotation. Prior to the simulations, hands-on practice had only occurred on the individual level in the department clinically with numbers ascertained prior to the simulation. A pre-survey had been filled out one month prior to the simulations.

The LAST immersive simulation was performed first for each participant to avoid participant bias and anchoring. For the simulation, participants were randomly divided into groups of approximately three residents for a 30-minute novel immersive case simulation with subsequent structured debrief and post-survey. The simulation was developed in conjunction with EM simulation fellowship-trained faculty. This case involved ascertaining a history and physical, which included a recent FICB for a traumatic hip fracture. The case progressed with the patient showing clinical signs and symptoms of LAST, including seizure and subsequent cardiac arrest. Participants were tested on and expected to develop a differential diagnosis for the patient’s presentation, identify LAST, and treat the patient with intralipid therapy as well as supportive care. The team was interdisciplinary with EM nurses and pharmacy residents also participating. Following the simulation during a debriefing session, diagnostic criteria and management of LAST were discussed using information provided by a board-certified toxicologist. Participants then filled out a post-survey evaluating their knowledge and comfort level regarding their recognition and management of LAST.

**Figure 1.**
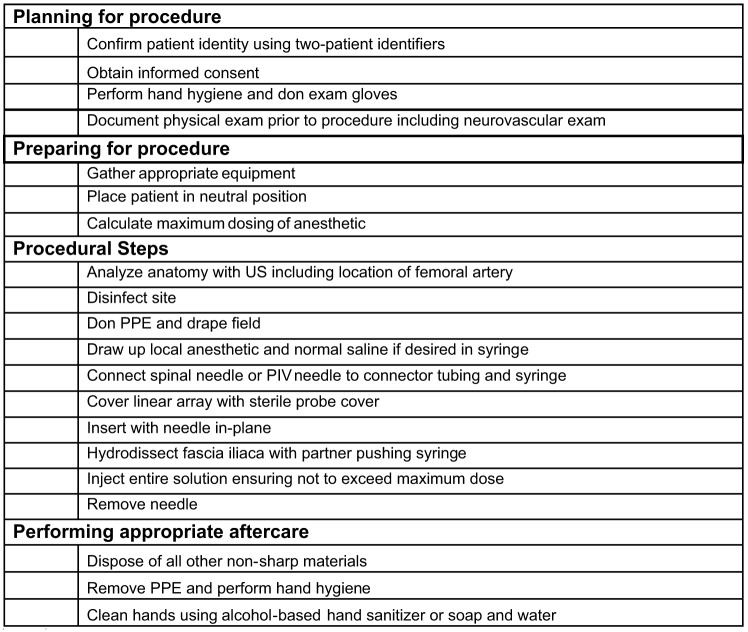
Learner checklist for fascia iliaca compartment block (FICB) procedure. *PPE*, personal protective equipment; *PIV*, peripheral intravenous; *US*, ultrasound.

Following completion of the LAST immersive simulation, participants were transitioned to the FICB procedural simulation in a separate location to avoid communication with incoming participants. Each group consisted of approximately six residents to allow for adequate hands-on time. A pre-survey was administered with specific questions directed toward residents’ perceived comfort and knowledge with the FICB procedure. A brief didactic lecture followed with focus on clinical indications for the procedure, US anatomy, procedural setup, and local anesthetic specifications and dosing. A reiteration of signs of LAST as well as management was included in the lecture as well. A standardized procedural checklist developed in conjunction with board-certified US faculty was used by all facilitators (Figure 1). Another handout for practicing calculating maximum doses was provided as a cognitive aid.

**Figure 2.**
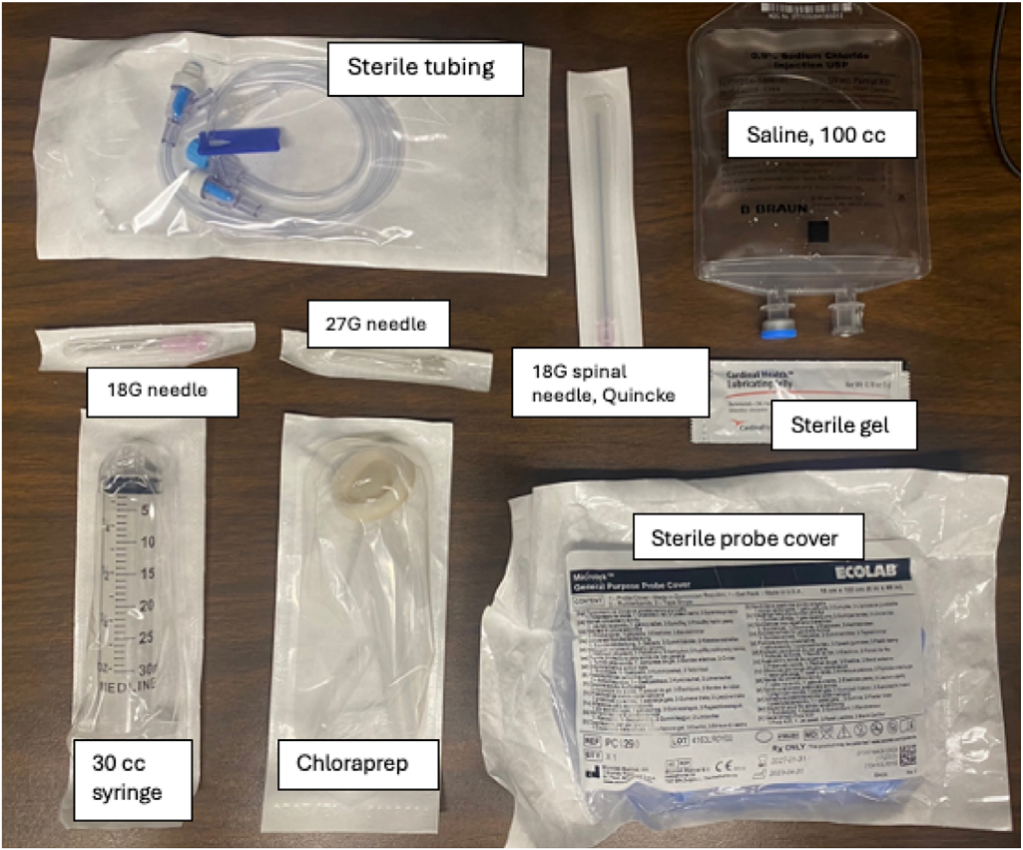
Supplies for fascia iliaca compartment block procedure. *G*, gauge; *cc*, cubic centimeter.

**Figure 3.**
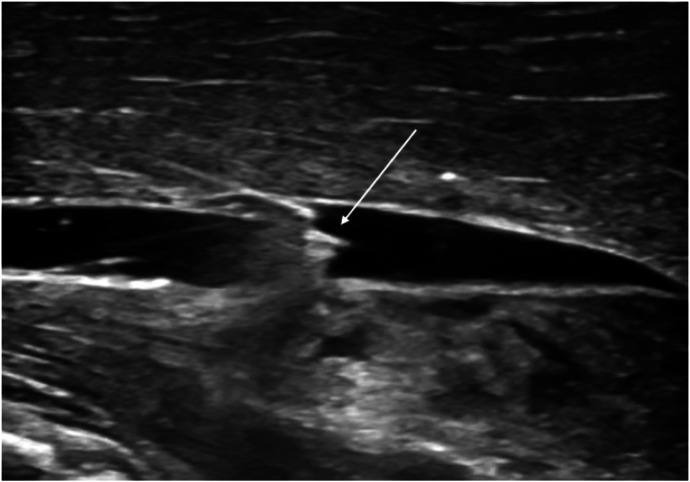
Simulation of hydrodissecting on porcine-tissue model (arrow indicating needle tip).

A standard setup of nerve block supplies was used (Figure 2). The procedural simulation used a fascia iliaca manikin (Valkyrie Simulators, Johnson Mills, WV) for practice visualizing anatomy with US, and a porcine-tissue model was used to practice hydrodissecting akin to performing the procedure in clinical practice (Figure 3). No manipulations were made to the porcine-tissue model including addition of mock nerve structures. Following completion of the procedural simulation, a post-survey was administered to the resident participants with specific questions pertaining to confidence and knowledge of procedural indications, relevant anatomy, and perceived comfort in performing the procedure following hands-on teaching and guidance. Board-certified US faculty directed all hands-on teaching and instruction.

We compared pre- and post-training survey responses using a generalized Stuart-Maxwell test to evaluate the impact of the training on residents’ knowledge and attitudes toward the procedure and LAST toxicity. Questionnaires included scale of 1–10 for FICB surveys and 1–7 for LAST surveys, with 1 corresponding to strongly disagree and the upper limits of 7 and 10 representing strongly agree, respectively. An alpha level of 0.05 was set for all statistical tests. We performed all analyses in SAS 9.4. (SAS Institute Inc, Cary, NC).^25^

Prior to implementation of both simulations, we performed walk-through sessions to anticipate and identify any logistical or systems issues. Nursing, pharmacy, and simulation staff were also consulted for input regarding the session as well as implementation in the department. A board-certified toxicologist also provided input to ensure management was consistent with the standard of care.

## RESULTS

A total of 19 EM residents ranging from PGY 1–3 participated in the LAST simulation, and 16 residents participated in the FICB procedural simulation. The distribution among training levels is described in Table 1 with a skew toward more PGY-3s (42.1% and 50%, respectively). Overall, 16 participants, or more than half (56%), had not received prior formal training on FICB. Half of the participants had previously performed a FICB, with the majority (31.3%) only performing 1–3 FICB prior to the session (Table 1). Following simulation, learners reported an improvement in confidence and knowledge with performing a FICB, with the pre-simulation improving from 4.0 (1.0–5.0) to 9.0 (7.0–10.0) post-simulation using the scale previously mentioned with 10 representing “strongly agree.” There was also an increase in the perception of the utility of FICB in the ED. Learners felt more confident in using US to visualize the relevant anatomy (4.0 [2.0–6.0] vs 9.0 [7.5–10.0]) and general knowledge of UGNB (5.0 [2.0–7.0] vs 10.0 [8.0–10.0]). Residents felt more confident in their ability to teach their peers the procedure (3.0 [1.0–5.0] vs 8.5 [7.0–10.0]). A general positive trend in comfort and knowledge was noted in the FICB following simulation, although no results were statistically significant (Table 2).

**Table 1.** Group characteristics.

Pre-simulation group characteristics	LAST (n = 19)	FICB (n = 16)
PGY level		
PGY-1	4 (21.1%)	3 (18.8%)
PGY-2	7 (36.8%)	5 (31.3%)
PGY-3	8 (42.1%)	8 (50%)
Prior to workshop: number of previously performed FICB		
I have never heard of it		0
0		8 (50%)
1–3		5 (31.3%)
4–6		1 (6.3%)
>6 times		2 (12.5%)

*PGY*, postgraduate year; *LQ*, lower quartile; *UQ*, upper quartile; *FICB*, fascia iliaca compartment block; *LAST*, local anesthetic systemic toxicity.

**Table 2.** Pre- and post-simulation reported experiences.

Mean reported pre- and post-simulation perceptions (FICB) (n = 16)	Median	LQ	UQ	*P*-value
Perceived knowledge of ultrasound-guided nerve blocks				
Pre-simulation	5.0	2.0	7.0	0.13
Post-simulation	10.0	8.0	10.0	
Perceived comfort level with performing FICB				
Pre-simulation	4.0	1.0	5.0	0.08
Post-simulation	9.0	7.0	10.0	
Perceived comfort visualizing fascia iliaca anatomy on US				
Pre-simulation	4.0	2.0	6.0	0.10
Post-simulation	9.0	7.5	10.0	
Perception of FICB utility in the ED				
Pre-simulation	8.0	7.0	10.0	0.13
Post-simulation	10.0	9.0	10.0	
Perceived comfort teaching procedure to peers				
Pre-simulation	3.0	1.0	5.0	0.20
Post-simulation	8.5	7.0	10.0	
**Mean reported pre- and post-simulation perceptions (LAST) (N = 19)**	**Median**	**LQ**	**UQ**	***P*-value**
Perceived confidence in diagnosing uncommon conditions				
Pre-simulation	5.0	4.0	6.0	0.79
Post-simulation	5.0	4.0	6.0	
Perceived confidence in managing uncommon conditions				
Pre-simulation	5.0	4.0	6.0	0.40
Post-simulation	5.0	4.0	6.0	
Perceived confidence in working in a multi-disciplinary team				
Pre-simulation	6.0	6.0	7.0	0.23
Post-simulation	6.0	5.0	7.0	
Perceived confidence in recognition of LAST				
Pre-simulation	5.0	3.0	6.0	0.12
Post-simulation	6.0	6.0	7.0	
Perceived confidence in management of LAST				
Pre-simulation	3.0	2.0	6.0	0.08
Post-simulation	6.0	6.0	7.0	

*PGY*, postgraduate year; *LQ*, lower quartile; *UQ*, upper quartile; *FICB*, fascia iliaca compartment block; *LAST*, local anesthetic systemic toxicity; *US*, ultrasound.

Learners rated similar perception in comfort with diagnosing and managing uncommon conditions both prior to and following the LAST simulation. There was an increase in comfort in both diagnosing and managing LAST. Recognition of LAST increased from 5.0 (3.0–6.0) to 6.0 (6.0–7.0) from pre- to post-simulation, respectively, with 7.0 representing strong agreement with a statement. Perception regarding management followed a similar trend with 3.0 (2.0–6.0) pre-simulation to 6.0 (6.0–7.0) post-simulation (Table 2), although neither was found to be statistically significant.

## DISCUSSION

Simulation is a key component of GME, for both immersive cases as well as practicing and mastering procedures, but there is limited research on combining both approaches to better mimic real-life practice and tie in connected topics. Simulation of the FICB, and its most serious adverse effect, LAST, offers a unique opportunity for resident simulation. With minimal resources and setup, it is possible for learners to experience the multitude of pathologic presentations and needed resuscitative measures of LAST. Although the main goal of both simulations was to assess perceptions in knowledge and confidence, the residents were also able to practice management of seizures, airway, and cardiac arrest while also fine-tuning US-guidance skills.

While our study was limited by underpowering, as is common in simulation-focused GME studies due to limited learner numbers, we found evidence of several important trends although they lacked statistical significance.^26^ In the timing immediately following simulation, residents reported an increasing trend in confidence and self-perceived knowledge, with the trend being more profound for the FICB procedural simulation. These findings are in line with prior procedural simulation-based research, demonstrating a trend toward improved learner confidence and knowledge following the procedure.^27^ ^,^ ^28^ Residents also noted increasing comfort with teaching peers the procedure. As resident perceptions were surveyed immediately following the simulation events, it is unclear whether those trends persisted beyond the day of simulation or were applied to subsequent clinical practice. Future investigations would benefit from follow-up at a scheduled interval and clinically focused outcome measures, such as procedural proficiency or the absence of adverse events.

Regarding the immersive simulation, no significant impact was seen in diagnosing and managing uncommon conditions or working in an interdisciplinary team, although there was a positive trend with residents’ ability to diagnose and manage LAST. Previous studies have focused on the impact of interprofessional teams for immersive simulation and have noted a positive correlation with appreciation and general knowledge of other healthcare professions.^29^ Despite resuscitation being a major component of EM training, this study may demonstrate a need for more detailed instruction regarding rarer causes of arrest.

Overall, perceptions of the simulation experience were also positive with learners indicating support of future sessions for other EM residents and clinicians. Although not statistically significant and largely similar pre- and post-simulation, the combination of cases was well received by residents in survey comments. Combination cases may provide a chance for a deeper grasp of integrated topics as well as a unique opportunity for residents to practice in an immersive simulation environment. The limited time available for resident education makes this approach valuable, as both simulations cover multiple aspects of medical care.

## LIMITATIONS

Limitations to the study include participants’ self-reported confidence and knowledge after a single encounter and, thus, may have been subject to bias given its subjective nature. While there was a general trend toward improvement in perceptions of knowledge and skills following the simulation, this is also in reference to short-term recall with further studies needed to ascertain long-term retention of knowledge and procedural skills. Further studies would also be required to elucidate the effects of combination simulation as a learning opportunity. Neither were we able to measure translation to real-world practice, with unclear integration of FICB into the participants’ future clinical practice. An expansion of the study with an extended timeline may show a decline in the knowledge and skills acquired in this simulation over time, as well as practice changes. Participation was another key limitation with a predominance of upper-level residents; and loss of participants may be attributed to scheduling difficulties particularly with off-service rotations.

Further limitations may exist if attempting to reproduce this experience at other sites; this simulation is based on faculty’s own perceived skills and confidence in performing, and teaching, FICB, which may not be as strong at other sites. This simulation may not be worthwhile at other sites if FICB procedures cannot be implemented given accessibility to intralipid emulsion therapy in case of possible LAST. Although research has already shown the benefit of using FICB in EDs both in the United States and resource-limited areas, further studies should be performed on teaching modalities, particularly for those with historically more limited ultrasound teaching.^13^ The homogenous responses in Table 3 may reflect further limitations to the study, which could reflect underlying bias.

**Table 3.** Learner perceptions regarding simulation experience.

Post-simulation learner experience survey	Median	LQ	UQ
The goals of the simulation were clearly outlined prior to participation	10.0	9.0	10.0
Felt had enough supervision during simulation	10.0	9.0	10.0
Felt comfortable asking questions or for help during the simulation	10.0	9.0	10.0
Felt was given adequate feedback during simulation	10.0	9.0	10.0
Simulation complimented learning style	10.0	9.0	10.0
This workshop would be useful for future ED residents and clinicians	10.0	9.0	10.0

*ED*, emergency department; *LQ*, lower quartile; *UQ*, upper quartile.

## CONCLUSION

Overall, this study continues to demonstrate the positive effect regarding the use of simulation in medical education, both with immersive and procedural simulations. Increasing exposure, even at an introductory level, of rarer pathology, including LAST, may aid in diagnosis and management. Residents’ perception of the procedural simulation in this study, while limited to fascia iliaca compartment block procedure, also had a positive trend toward comprehension and skillset. Although limited in the number of participants, this study demonstrates that the use of combination immersive and procedural simulation may provide an exciting and worthwhile experience for learners, particularly with interconnected topics.
